# Prevalence of Periodontitis and Methodological Aspects of the 2018 EFP/AAP Classification—The Study of Health in Pomerania (SHIP‐TREND)

**DOI:** 10.1111/jcpe.70137

**Published:** 2026-04-29

**Authors:** Sonya Nafz, Thomas Kocher, Christiane Pink, Henry Völzke, Philipp Kanzow, Birte Holtfreter

**Affiliations:** ^1^ Department of Restorative Dentistry, Periodontology and Endodontology University Medicine Greifswald Greifswald Germany; ^2^ Institute for Community Medicine, SHIP/Clinical‐Epidemiological Research University Medicine Greifswald Greifswald Germany; ^3^ DZHK (German Center for Cardiovascular Research), partner siteGreifswald Greifswald Germany

**Keywords:** ACES framework, CDC/AAP classification, cohort study, EFP/AAP classification, periodontitis, tooth loss

## Abstract

**Aim:**

To estimate the prevalence of periodontitis and highlight methodological issues using the Application of the 2018 Periodontal Status Classification to Epidemiological Survey Data (ACES) framework.

**Materials and Methods:**

We used data from the population‐based Study of Health in Pomerania (SHIP‐TREND; age 20–84 years). Periodontal status was recorded using a half‐mouth protocol. We estimated the prevalence of periodontitis, evaluated changes in the assignment of stages to combinations of available complexity factors, associated periodontal status with 7‐year tooth loss and illustrated agreement with the Centers for Disease Control and Prevention/American Academy of Periodontology (CDC/AAP) classification.

**Results:**

The total prevalence of periodontitis was 78.6%; 18.0% and 17.4% were Stage III and Stage IV, respectively. Grade C was exhibited by 49.1% of Stage III cases and 37.8% of Stage IV cases. Assignment of stage changed only marginally when complexity factors were considered in addition to interdental clinical attachment levels. Three percent of Stage IV cases and 15.2% of non‐classified cases became toothless. Adjusted incidence rates for tooth loss were 3.60 for Stage III cases, 6.55 for non‐classified cases and 8.69 for Stage IV cases. Severe cases (CDC/AAP) corresponded primarily to Stage III (42.9%) and Stage IV (53.0%) cases.

**Conclusions:**

For public health purposes, caution should be exercised when translating the periodontitis prevalence according to the ACES framework into treatment requirements. Furthermore, complex and effective interventions are required for non‐classified and Stage IV cases in order to prevent tooth loss and edentulism.

## 1 Introduction

Periodontitis is a multifactorial chronic inflammatory disease affecting the supporting structures of the teeth. The prevalence of periodontitis varies considerably on a global scale (Nascimento et al. 2024). This is due to variations in diagnostic methodology and the use of different thresholds and case definitions (Holtfreter et al. 2015), as well as socioeconomic, behavioural, genetic, environmental and public dental care factors. Following the development of the European Federation of Periodontology/American Academy of Periodontology (EFP/AAP) classification (Papapanou et al. 2018; Tonetti et al. 2018), the Application of the 2018 Periodontal Status Classification to Epidemiological Survey Data (ACES) framework was developed to standardise its application and guide data collection in epidemiological studies (Holtfreter et al. 2024). It also addresses the limitations of the 2018 EFP/AAP classification, such as its reliance on clinical judgement for staging and grading, which are impractical in epidemiological research.

Representative national and regional periodontitis prevalence data using the EFP/AAP classification with or without the application of the ACES framework have already been reported for mainland China (Jiao et al. 2021), the United States (Meng et al. 2025; Tay et al. 2025), the county of Nord‐Trøndelag, Norway (Stodle et al. 2021), Tromsø, Norway (Holde et al. 2025), a rural area of Rosário do Sul, Brazil (Ortigara et al. 2021), the city of Curitiba, Brazil (Dos Anjos et al. 2024), Chile (Morales et al. 2022), and Germany (Eickholz et al. 2025), with Stages III–IV prevalences ranging between 17% and 95% (Table S1). However, data for the entire adult age range in Germany, using the ACES framework, are lacking.

Using the 2018 EFP/AAP classification in combination with the ACES framework poses several methodological challenges. Firstly, prevalence estimates in Germany exceeding 80% raise concerns of potential overestimation of treatment needs (Eickholz et al. 2025). Secondly, it is important to evaluate whether the omission of complexity factors (Ortigara et al. 2021; Holde et al. 2025) may have resulted in biased periodontitis prevalence estimates. Consequently, the question arises as to whether the ACES framework can be simplified, and if so, to what extent. Thirdly, non‐classified cases require closer consideration, since they were not captured by the original EFP/AAP classification due to a definitional gap (Holtfreter et al. 2024). These cases must be characterised in terms of disease severity, tooth condition and risk of tooth loss. Fourthly, it is necessary to validate the plausibility of the ACES framework in relation to tooth loss. In this regard, it was hypothesised that increased tooth loss in Stage IV leads to bite collapse (Tonetti et al. 2018). Fifthly, levels of agreement with the CDC/AAP classification (Eke et al. 2012) were reported as ranging from moderate (Dos Anjos et al. 2024; Eickholz et al. 2025; Tay et al. 2025) to high (Ortigara et al. 2021). Obviously, the disparities between the classifications are primarily influenced by the basic threshold for diagnosing periodontitis.

Consequently, the overarching aims of this work are two‐fold. The primary objective is to estimate the prevalence of periodontitis in the Study of Health in Pomerania (SHIP‐TREND) using the ACES framework. The secondary objective is to examine the methodological challenges of the ACES framework, which encompass (i) differentiation between estimated prevalence and actual needs for treatment; (ii) relevance of the complexity factors; (iii) describing non‐classified cases in terms of risk of tooth loss; (iv) association of the ACES framework with 7‐year tooth loss; and (v) agreement in prevalence estimates with the CDC/AAP classification.

## 2 Material and Methods

### 2.1 Study Design

SHIP‐TREND is a population‐based cohort study conducted in West Pomerania, Germany (Völzke et al. 2022). A stratified random sample (based on age, sex and city/county of residence) of 10,000 adults aged 20–79 years was drawn from the central population registries of Mecklenburg‐Western Pomerania. The target sample size was chosen to obtain a final sample size similar to that of the SHIP‐START‐0 cohort study, which was conducted in the same region 10 years ago (Völzke et al. 2022). After excluding migrants (*N* = 851) and deceased individuals (*N* = 323), the net sample consisted of 8826 individuals. Because of several reasons (241 did not answer, 3367 refused participation, 549 did not keep the appointment and 249 agreed without an appointment), 4420 subjects were finally recruited in the baseline study SHIP‐TREND‐0 (2008–2012; response 50.1%). Of those, 2507 participants completed the 7‐year follow‐up (see Figure S1; SHIP‐TREND‐1; 2016–2019), with an average follow‐up of 7.4 years.

The reporting followed the recommendations of the Strengthening the Reporting of Observational Studies in Epidemiology (STROBE) guidelines for observational studies (von Elm et al. 2014).

The Supporting Information contains detailed information on periodontal examinations, definitions of periodontal variables and classifications, calibration data and covariates.

### 2.2 Statistical Analyses

Means and standard deviations (SDs) as well as medians with 25% and 75% quantiles were reported for continuous variables. Relative frequency distributions were computed for categorical variables. First, we presented the prevalence data using the ACES framework for completed studies, both in total and according to age and sex. Owing to the complex sample design, we calculated standard errors (SEs) for total prevalence estimates, considering design variables (strata identification) and sampling weights. For periodontitis cases, we also reported the grade and extent. Sex differences in the distribution according to the ACES framework were tested using chi‐squared (*χ* ^2^) tests. Second, we determined stage using different combinations of severity (interdental CAL) and two out of eight complexity factors (PD and the number of opposing pairs of natural teeth). Third, we correlated the periodontal status according to the ACES framework with incident tooth loss using 7‐year follow‐up data. We reported the number of teeth at baseline, the number of extracted teeth, follow‐up time (in exact years) and the number of incidentally edentulous persons. On the tooth level (full‐mouth), the number of extracted teeth was determined. Furthermore, negative binomial regression models with robust SEs adjusted for age, sex, education, smoking, known diabetes, haemoglobin A1c, body mass index and follow‐up time (log; offset) were evaluated. The continuous variables were modelled linearly following the inspection of non‐linear terms. Models were weighted using inverse probability weights to account for differential loss to follow‐up. Incidence rate ratios (IRRs) with 95% confidence intervals (CIs) were reported. Fourth, Sankey plots were used to visualise transitions between the CDC/AAP classification and the ACES framework. We also provided diagnostic performance measures, including the level of agreement (with SEs), Cohen's κ, sensitivity, specificity, and positive (PPV) and negative predictive values (NPV; all with 95% CIs) for identifying (i) moderate to severe cases (CDC/AAP), using Stages III–IV cases (ACES framework), and (ii) severe cases (CDC/AAP) using Stage IV cases (ACES framework).

A two‐sided *p* < 0.05 was considered statistically significant. All analyses were performed using Stata/SE Version 17.0 (StataCorp 2021).

## 3 Results

### 3.1 Baseline Characteristics

Participants included in cross‐sectional analyses had an average age of 51.3 years, 49.2% were male, 26.5% had more than 10 years of school education, 26.8% were current smokers, 25.3% used powered toothbrushes alone or in combination with manual toothbrushes and 26.7% reported using interdental cleaning aids (see Table 1). Averages for BOP, mean PD and mean interdental CAL were 25.3%, 2.59 mm, and 2.56 mm, respectively. Seventeen percent had severe periodontitis according to the CDC/AAP classification. Detailed descriptive data stratified by periodontitis status are provided in Table S2.

**TABLE 1:** Baseline characteristics of participants included in the cross‐sectional (*N* = 3890) and longitudinal analyses (*N* = 2294).

	*N*	Cross‐sectional analyses (SHIP‐TREND‐0)	*N*	Longitudinal analyses (SHIP‐TREND‐0 and SHIP‐TREND‐1)
Follow‐up time, years	—	—	2294	7.4 ± 0.7 7.3 (7.0; 7.5)
Age, years	3890	51.3 ± 15.2 52 (39; 63)	2294	49.2 ± 13.6 49 (39; 59)
Male sex	3890	1915 (49.2%)	2294	1111 (48.4%)
School education	3882		2289	
< 10 years		833 (21.5%)		283 (12.4%)
10 years		2021 (52.1%)		1272 (55.6%)
> 10 years		1028 (26.5%)		734 (32.1%)
Living in a partnership, yes	3882	3031 (78.1%)	2289	1856 (81.1%)
Smoking status	3881		2289	
Never		1410 (36.3%)		891 (38.9%)
Former		1430 (36.9%)		873 (38.1%)
Current, < 10 cigarettes/day		218 (5.6%)		132 (5.8%)
Current, ≥ 10 cigarettes/day		701 (18.1%)		312 (13.6%)
Current, missing		122 (3.1%)		81 (3.5%)
Body mass index, kg/m^2^	3885	28.0 ± 5.1 27.4 (24.4; 30.9)	2292	27.4 ± 4.6 27.0 (24.2; 30.1%)
Haemoglobin A1c, %	3883	5.3 ± 0.8 5.2 (4.9; 5.6)	2290	5.3 ± 0.7 5.2 (4.8; 5.6)
Known type 2 diabetes mellitus, yes	3885	378 (9.7%)	2291	128 (5.6%)
Haemoglobin A1c ≥ 7% in individuals with known type 2 diabetes, yes	378	121 (32.0%)	128	36 (28.1%)
Daily use of interdental cleaning aids, yes	3830	1023 (26.7%)	2294	672 (29.3%)
Tooth brushing frequency	3830		2294	
< 2 times daily		593 (15.5%)		290 (12.6%)
≥ 2 times daily		3237 (84.5%)		2004 (87.4%)
Tooth brush type	3830		2294	
Neither MTB nor PTB		43 (1.1%)		9 (0.4%)
MTB		2816 (73.5%)		1623 (70.8%)
PTB		740 (19.3%)		501 (21.8%)
MTB and PTB		231 (6.0%)		161 (7.0%)
Dental visit within the last 12 months, yes	3830	3402 (88.8%)	2294	2106 (91.8%)
Self‐reported periodontal treatment within the last 5 years, yes	3806	703 (18.5%)	2.282	461 (20.2%)
Percentage of sites with bleeding on probing, %	3618	25.3 ± 24.4 18.8 (6.3; 37.5)	2291	23.6 ± 23.2 16.7 (5.0; 33.3)
Mean PD, mm	3621	2.59 ± 0.71 2.40 (2.14; 2.83)	2294	2.53 ± 0.62 2.38 (2.13; 2.73)
Percentage of sites with PD ≥ 4 mm, %	3621	14.5 ± 19.4 6.3 (0; 20.5)	2294	12.7 ± 17.4 5.4 (0; 17.9)
Mean CAL, mm	3432	2.47 ± 1.70 2.07 (1.27; 3.33)	2198	2.30 ± 1.53 1.96 (1.23; 3.02)
Mean interdental CAL, mm	3430	2.56 ± 1.80 2.17 (1.29; 3.50)	2196	2.38 ± 1.65 2.05 (1.21; 3.18)
CDC/AAP classification	3347		2161	
No periodontitis		1337 (40.0%)		901 (41.7%)
Mild periodontitis		279 (8.3%)		188 (8.7%)
Moderate periodontitis		1167 (34.9%)		746 (34.5%)
Severe periodontitis		564 (16.8%)		326 (15.1%)
Number of teeth	3890	20.2 ± 8.6 24 (17; 27)	2294	22.6 ± 6.1 25 (21; 27)

*Note*: Data are presented as means ± standard deviations and medians (25%; 75% quantiles) or as numbers (percentages).

Abbreviations: AAP, American Academy of Periodontology; CAL, clinical attachment level; CDC, Centers for Disease Control and Prevention; MTB, manual tooth brush; PD, probing depth; PTB, powered tooth brush.

For longitudinal analyses, participants were followed, on average, over 7.4 years. On average, the respondents were 49.2 years old at baseline. Of these, 32.1% had attended school for more than 10 years, 22.9% were current smokers, and 29.3% reported using interdental cleaning aids.

### 3.2 Prevalence of Gingivitis and Periodontitis according to the EFP/AAP Classification

Total as well as age‐ and sex‐stratified prevalences of gingivitis and periodontitis were determined according to the ACES framework (Table 2; see Table S3 for estimates in 10‐year age groups starting at < 25 years). Overall, 6.7% of the participants were edentulous, 3.0% were periodontally healthy, 2.7% had gingivitis, 9.0% were non‐classified and 78.6% had periodontitis which was further subdivided into 15.0% Stage I, 28.2% Stage II, 18.0% Stage III and 17.4% Stage IV. Grade A was found in 3.6% of Stage I cases (Table 3). Grade C was exhibited by 49.1% of Stage III cases and 37.8% of Stage IV cases. Refer to Table S4 for grades according to Schumacher and Gente (1995). Generalised periodontitis predominated in Stage I (93.8%) and Stage IV (73.0%).

**TABLE 2:** Distribution of SHIP‐TREND‐0 participants (*N* = 3890) according to the ACES framework in total, as well as by sex and age group.

	Total	Females	Males	20–29 years	30–39 years	40–49 years	50–59 years	60–69 years	70–84 years
Edentulous	6.7% (0.38)	6.8% (0.58)	6.6% (0.50)	0% (−)	0% (−)	0.6% (0.25)	5.7% (0.80)	10.8% (1.21)	26.4% (2.01)
Periodontal health	3.0% (0.32)	3.6% (0.50)	2.3% (0.40)	13.4% (1.92)	4.6% (0.88)	1.4% (0.40)	0.24% (0.17)	0.1% (0.10)	0% (−)
Localised gingivitis	2.2% (0.27)	2.0% (0.35)	2.5% (0.41)	9.2% (1.58)	3.6% (0.75)	1.0% (0.34)	0.55% (0.28)	0.4% (0.21)	0% (−)
Generalised gingivitis	0.5% (0.14)	0.5% (0.18)	0.6% (0.20)	1.5% (0.71)	1.0% (0.44)	0.1% (0.15)	0.5% (0.23)	0.2% (0.17)	0% (−)
Periodontitis cases	78.6% (0.65)	76.7% (0.97)	80.4% (0.88)	75.7% (2.37)	90.3% (1.21)	94.2% (0.83)	81.8% (1.34)	70.4% (1.74)	50.1% (2.28)
Stage I	15.0% (0.59)	16.8% (0.84)	13.3% (0.82)	40.0% (2.71)	32.4% (2.01)	13.5% (1.23)	5.8% (0.81)	2.6% (0.59)	0.7% (0.38)
Stage II	28.2% (0.73)	28.8% (1.01)	27.6% (1.06)	30.4% (2.49)	42.2% (2.10)	40.6% (1.75)	26.5% (1.55)	16.7% (1.38)	7.7% (1.19)
Stage III	18.0% (0.61)	15.0% (0.81)	20.8% (0.92)	5.3% (1.22)	13.1% (1.36)	28.0% (1.63)	24.2% (1.51)	19.5% (1.47)	9.8% (1.35)
Stage IV	17.4% (0.58)	16.0% (0.82)	18.8% (0.83)	0% (−)	2.6% (0.72)	12.1% (1.20)	25.3% (1.52)	31.6% (1.78)	31.9% (2.13)
Non‐classified cases	9.0% (0.44)	10.5% (0.69)	7.6% (0.56)	0.2% (0.20)	0.5% (0.24)	2.7% (0.60)	11.2% (1.10)	18.1 (1.48)	23.5% (1.94)
Total number of subjects	3890 (100%)	1975 (100%)	1915 (100%)	355 (100%)	619 (100%)	805 (100%)	840 (100%)	743 (100%)	528 (100%)

*Note*: Data are given as percentages with standard errors in brackets. Survey‐weighted estimates are reported.

Abbreviation: ACES, Application of the 2018 Periodontal Status Classification to Epidemiological Survey Data.

**TABLE 3:** Grade (according to Salonen et al.) and extent in periodontitis cases according to the ACES framework.

Stage	*N*	Grade			Extent	
		Grade A	Grade B	Grade C	Localised	Generalised
Stage I	522	3.6% (0.75)	81.8% (1.79)	14.6% (1.68)	6.2% (0.99)	93.8% (0.99)
Stage II	1104	0% (−)	75.8% (1.38)	24.2% (1.38)	52.8% (1.57)	47.2% (1.57)
Stage III	729	0% (−)	50.9% (1.89)	49.1% (1.89)	60.0% (1.88)	40.0% (1.88)
Stage IV	716	0% (−)	62.2% (1.86)	37.8% (1.86)	27.0% (1.73)	73.0% (1.73)

*Note*: Data are given as percentages with standard errors in brackets. Survey‐weighted estimates are reported.

Abbreviation: ACES, Application of the 2018 Periodontal Status Classification to Epidemiological Survey Data.

Although there were statistically significant sex differences in the distribution according to the ACES framework (*p* < 0.001), the differences in total periodontitis prevalence was negligible (76.7% vs. 80.4%). The sex difference in the prevalence of Stage III (15.0% in females vs. 20.8% in males) and Stage IV (16.0% in females vs. 18.8% in males) was more obvious. Across all age groups, the proportion of staged periodontitis cases increased up to the age of 40–49 years and then decreased in favour of the non‐classified and edentulous (with proportions reaching 23.5% and 26.4%, respectively, in the oldest age group). After the age of 60, the most common stages of periodontitis were III and IV.

### 3.3 Influence of Complexity Factors on the Staging Process

We evaluated the influence of complexity factors considered in addition to maximum interdental CAL (severity factor) on the distribution of stages in periodontitis cases (Table 4). When maximum interdental CAL was the only determinant of staging, 522 cases were classified as Stage I, 1118 as Stage II and 1431 as Stage III/IV. Differentiation between stages III and IV was not possible. When PD was additionally considered to differentiate between stages II and III (second line), only 14 cases shifted from Stage II to Stage III. Accounting only for the number of opposing pairs of natural teeth allowed for the differentiation between Stages III and IV (1431 Stage III/IV cases were split into 715 Stage III cases and 716 Stage IV cases). Combining information from PDs and the number of opposing pairs of natural teeth (fourth line) resulted in the shift of 14 Stage II cases to Stage III compared to when no complexity factors were considered.

**TABLE 4:** Consideration of different complexity factors in addition to maximum interdental CAL (severity factor) in the staging process, applying the ACES framework (*N* = 3071 periodontitis cases).

Considered complexity factors	Stage I	Stage II	Stage III/IV	Stage III	Stage IV
No complexity factors considered	522 (19.1%)	1118 (36.4%)	1431 (44.5%)	—	—
PD ≥ 6 mm at ≥ 2 non‐adjacent teeth	522 (19.1%)	1104 (35.9%)	1445 (45.0%)	—	—
< 10 opposing pairs of natural teeth	522 (19.1%)	1118 (36.4%)	—	715 (22.4%)	716 (22.2%)
All complexity factors (PD ≥ 6 mm at ≥ 2 non‐adjacent teeth, < 10 opposing pairs of natural teeth)	522 (19.1%)	1104 (35.9%)	—	729 (22.9%)	716 (22.2%)

*Note*: Data are given as numbers (unweighted) with percentages (survey‐weighted) in brackets.

Abbreviations: ACES, Application of the 2018 Periodontal Status Classification to Epidemiological Survey Data; CAL, clinical attachment level; NA, not available; PD, probing depth.

### 3.4 Associations With 7‐Year Tooth Loss

The associations between periodontal status according to the ACES framework and 7‐year tooth loss are presented in Table 5. The average number of teeth at baseline was highest among periodontally healthy individuals (26.7 ± 2.3) and lowest among non‐classified cases (8.7 ± 5.9). Among those with Stage IV periodontitis, 2.6% became edentulous (Stage I and Stage III: 0%; Stage II: 0.3%), while 15.2% of non‐classified cases were affected. Compared to individuals with periodontal health or gingivitis, adjusted incidence rates increased steadily from 1.37 (Stage I) to 8.69 (Stage IV). For non‐classified cases, incidence rate was nearly seven‐fold (IRR = 6.55; 95% CI: 3.71–11.55).

**TABLE 5:** Tooth loss over 7 years (full mouth) in relation to ACES framework status (*N* = 2294).

		Subject level data				Tooth level data	Subject level data (*N* = 2284)	
ACES framework	Number of subjects	Number of teeth at baseline	Number of extracted teeth	Follow‐up time, years	Number (%) of incidentally edentulous persons	Number (%) of extracted teeth	Crude IRRs (95% CI)^a^	Fully adjusted IRRs (95% CI)^a^
Periodontal health	61	26.7 ± 2.3 28 (26; 28)	0.15 ± 0.36 0 (0; 0)	7.23 ± 0.63 7.19 (6.98; 7.40)	0 (0%)	9 (0.6%)	1.00 (ref.)	1.00 (ref.)
Gingivitis	48	26.2 ± 2.8 28 (25; 28)	0.21 ± 0.58 0 (0; 0)	7.24 ± 0.79 7.13 (6.90; 7.48)	0 (0%)	9 (0.6%)		
Stage I	360	26.1 ± 2.5 27 (25; 28)	0.24 ± 0.60 0 (0; 0)	7.36 ± 0.65 7.28 (7.03; 7.48)	0 (0%)	84 (0.9%)	1.37 (0.78; 2.39)	1.37 (0.79; 2.36)
Stage II	757	24.6 ± 3.3 25 (23; 27)	0.60 ± 1.34 0 (0; 1)	7.35 ± 0.67 7.26 (7.03; 7.50)	2 (0.3%)	451 (2.4%)	3.74 (2.21; 6.32)	2.70 (1.62; 4.49)
Stage III	505	25.4 ± 1.9 26 (24; 27)	0.95 ± 1.84 0 (0; 1)	7.38 ± 0.60 7.28 (7.05; 7.49)	0 (0%)	479 (3.7%)	5.51 (3.28; 9.27)	3.60 (2.12; 6.11)
Stage IV	385	17.2 ± 4.7 18 (14; 21)	2.75 ± 3.60 1 (0; 4)	7.47 ± 0.65 7.31 (7.12; 7.55)	10 (2.6%)	1065 (16.1%)	15.69 (9.44; 26.09)	8.69 (5.08; 14.85)
Non‐classified cases	178	8.7 ± 5.9 8 (4; 12)	2.15 ± 2.58 1 (0; 3)	7.43 ± 0.69 7.29 (7.02; 7.51)	27 (15.2%)	386 (25.0%)	12.64 (7.46; 21.42)	6.55 (3.71; 11.55)
Total population	2294	22.6 ± 6.1 25 (21; 27)	1.08 ± 2.21 0 (0; 1)	7.38 ± 0.65 7.28 (7.04; 7.50)	39 (1.7%)	2483 (4.8%)	1.26 (1.15; 1.37)	—

*Note*: Data are presented as means ± standard deviations and medians (25%; 75% quantiles) or numbers (percentages).

Abbreviations: ACES, Application of the 2018 Periodontal Status Classification to Epidemiological Survey Data; IRR, incidence rate ratios.

^a^ Retrieved from negative binomial regression models with robust standard errors. Adjustment included age (continuously), sex, school education, smoking status, known diabetes, haemoglobin A1c (continuously), body mass index (continuously) and follow‐up time (log; offset).

### 3.5 Comparison of the ACES Framework With the CDC/AAP Classification

**FIGURE 1:**
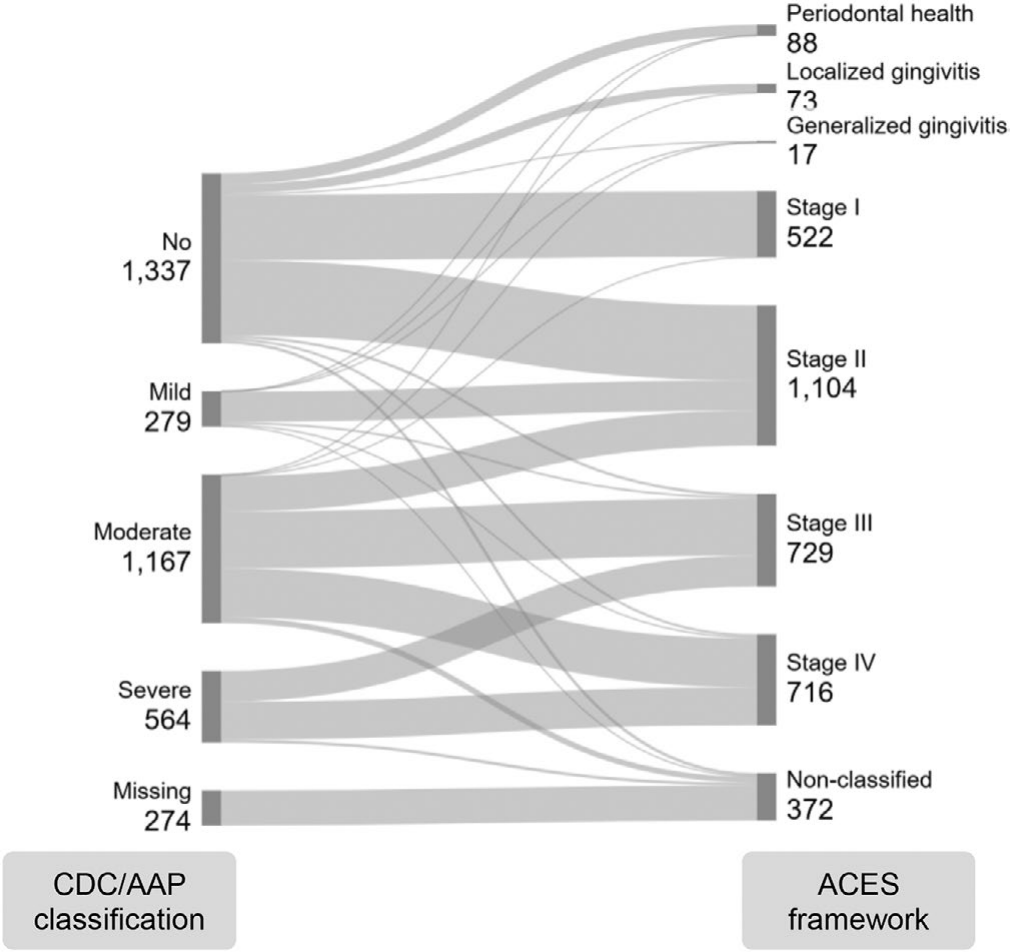
Sankey graph illustrating the transitions between the CDC/AAP classification and the ACES framework. Edentulous persons were excluded from the graph. For the CDC/AAP classification, individuals were categorised as non‐classified if they had < 2 teeth with CAL measurements or < 2 teeth with PD measurements. AAP, American Academy of Periodontology; ACES, Application of the 2018 Periodontal Status Classification to Epidemiological Survey Data; CDC, Centers for Disease Control and Prevention.

Using the Sankey graphs (Figure 1, Table S5), with a detailed breakdown of the categories, we observed a low degree of agreement between the CDC/AAP classification and the ACES framework. Of those classified as having no periodontitis by the CDC/AAP classification, 38.6% were classified as Stage I and 44.1% were classified as Stage II. Moderate periodontitis exhibited partial correspondence with Stage II (23.6%), but predominantly transitioned into Stages III (38.5%) and IV (32.9%). Severe periodontitis cases predominantly transitioned into Stages III (42.9%) and IV (53.0%).

However, when the categories were merged to determine the diagnostic performance measures of the ACES framework compared to the CDC/AAP classification (Table S6), substantial agreement was found (88.9%), with a Cohen's κ of 0.78 for identifying cases of moderate to severe periodontitis using the ACES framework (i.e., opposing ‘Stage III/IV’ vs. ‘Healthy to Stage II’). Having Stage III to IV periodontitis (ACES framework) was highly sensitive in identifying individuals with moderate to severe periodontitis (CDC/AAP), with a sensitivity of 95.1% (95% CI: 93.8%–96.1%) and a specificity of 84.0% (82.2%–85.6%). The PPV and NPV were 82.6% (80.7%–84.4%) and 95.5% (94.4%–96.5%), respectively.

However, the agreement decreased to 79.7% for the identification of severe cases (CDC/AAP) by the ACES framework (opposing ‘Stage IV’ vs. ‘Healthy to Stage III’), and Cohen's κ was only fair (0.353; SE = 0.017). The sensitivity for identifying severe cases (CDC/AAP) decreased to 41.8% (95% CI: 38.1%–45.5%) with a specificity of 90.4% (89.2%–91.6%). The PPV and NPV were 55.3% (51.0%–59.5%) and 84.6% (83.2%–85.9%), respectively. This was already evident from the Sankey graphs (Figure 1).

## 4 Discussion

In SHIP‐TREND‐0, the prevalence of periodontitis according to the EFP/AAP classification when using the ACES framework was 78.6%, with 15.0% classified as Stage I, 28.2% as Stage II, 18.0% as Stage III and 17.4% as Stage IV periodontitis. Sex‐based disparities in Stage III and Stage IV prevalences emerged (15.0% and 16.0% in females vs. 20.8% and 18.8% in males, respectively). Also, age‐based disparities were evident: the prevalence of periodontitis increased from 75.7% in individuals aged 20–29 to 94.2% in individuals aged 40–49, before declining to 50.1% in individuals aged 70–84 due to an increasing prevalence of edentulism (26.4%) and non‐classified cases (23.5%).

Although the overall prevalence was 78.6%, comparisons with other studies are limited by SHIP‐TREND's partial recording protocol, which underestimates disease compared to full‐mouth protocols (see Table S1). Nevertheless, the following section presents an overview of the studies with EFP/AAP classification data that have been published to date. At the national level (DMS **•** 6), periodontitis prevalence was comparable for individuals aged 35–44 (93.7% vs. 95.1%) but lower in individuals aged 65–74 (60.3% vs. 85.2%) (Eickholz et al. 2025). In the HUNT4 study in Norway (2017–2019), a slightly lower prevalence of periodontitis was observed in individuals aged 19–94 (72.4%), with 15.3% in Stage III and 2.3% in Stage IV (Stodle et al. 2021). In the United States (NHANES; 2009–2014; age ≥ 30 years), the prevalence of periodontitis was higher (93.2%) than in SHIP‐TREND‐0 (78.6%), with comparable prevalences of Stage III (16.7% vs. 18.0% in SHIP‐TREND‐0) and a lower prevalence of Stage IV periodontitis (12.4% vs. 17.4% in SHIP‐TREND‐0) being reported (Tay et al. 2025). Compared to data from mainland China (Fourth National Oral Health Survey), the total periodontitis prevalence in SHIP‐TREND‐0 was higher in individuals aged 35–44 (93.2% vs. 52.8%) but similar in individuals aged 65–74 (62.4% vs. 64.6%) (Jiao et al. 2021). However, the prevalence of Stages III/IV was consistently higher in SHIP‐TREND‐0 in all age groups. In the study from Curitiba, Brazil (Dos Anjos et al. 2024), prevalences of Stage III (24.0%) and Stage IV (19.3%) periodontitis in adults aged ≥ 18 years were slightly higher than in SHIP‐TREND‐0. Finally, in a study of individuals aged ≥ 60 from the Philippines, higher overall (100%) and Stage III/IV prevalences (94.5%) were observed (Garcia et al. 2024). In summary, considering the SHIP‐TREND‐0 partial recording protocol, prevalences of Stage III/IV periodontitis in SHIP‐TREND‐0 might be higher than for Norway, the United States and China, but still lower than for Brazil, Chile and the Philippines.

In terms of grading, 49.1% of Stage III cases and 37.8% of Stage IV cases in SHIP‐TREND‐0 exhibited Grade C. This contrasts with findings from Stodle et al. (2021), where only 5.3% of Stage III and 23.4% of Stage IV cases were classified as Grade C. In the study from Ciritiba, Brazil, 52.5% of Stage III and 52.3% of Stage IV cases had Grade C (Dos Anjos et al. 2024). However, Grade B was the predominant grade in all three studies. Furthermore, Grade A was observed only in 3.6% of Stage I cases in SHIP‐TREND‐0. This can be explained by the EFP/AAP classification criteria, since the limited cumulative bone loss characteristic of Stage I generally results in a low bone loss‐to‐age ratio, whereas the greater extent of historical bone destruction and the more frequent presence of risk factors in advanced stages reduce the likelihood of a Grade A assignment.

In terms of methodological challenges, the following results were observed: (i) The ACES framework resulted in a comparably high periodontitis prevalence; (ii) Interdental CAL dominated the staging procedure; (iii) Non‐classified cases presented with comparably high tooth loss rates and the highest proportion of incidentally edentulous cases (15.2%), presenting a distinct, until now undescribed group; (iv) Compared to Stage III cases, Stage IV cases presented a higher proportion of incidental edentulism (2.6%) and a higher proportion of extracted teeth (16.1% vs. 3.7%); (v) While agreement between the CDC/AAP and the ACES framework (with consideration of all single categories) was low (Figure 1), Stage III/IV cases corresponded well to moderate to severe periodontitis cases (CDC/AAP).

Firstly, it is crucial to accurately estimate the prevalence of conditions requiring treatment in order to allocate dental healthcare resources effectively. In SHIP‐TREND‐0, the prevalence of Stages I–IV periodontitis was estimated to range from 50.1% to 94.2%, depending on age, using a half‐mouth protocol. Converting to full‐mouth estimates would result in even higher prevalences. However, the national DMS * 6 study reported similarly high estimates: periodontitis was present in 95.1% of individuals aged 35–44 and 85.2% of individuals aged 65–74 (Eickholz et al. 2025). These high prevalences may be partly explained by misclassification as Stage I periodontitis rather than gingivitis. This could be due to an overestimation of CAL in sites with gingival inflammation, as well as variability in CAL measurements of ±1 mm, particularly when the cemento‐enamel junction (CEJ) is obscured (Tay et al. 2025). In such cases, incorporating radiographic imaging could increase diagnostic precision, as radiographically detectable alveolar bone loss would allow for a more accurate classification.

However, when prevalence estimates are translated into treatment needs, we should question whether subgingival instrumentation, supplemented by adjunctive interventions, as well as re‐evaluation, is required for all patients at all stages (Sanz et al. 2020), or whether this should be allocated more specifically. In this regard, Stage I periodontitis has been suggested as representing a transitional phase between gingivitis and early periodontitis (Eickholz et al. 2025), which is likely to be managed through improved home and professional hygiene care, as well as risk factor control, rather than subgingival instrumentation. Such considerations are particularly important when planning treatment times and expenditures.

Secondly, in SHIP‐TREND‐0, interdental CAL was the predominant factor in the staging process. Complexity factors such as PD and the number of opposing pairs of natural teeth were somewhat less influential. When PDs were considered to differentiate between stages II and III, only 14 cases (0.5%) were reclassified to Stage III. Considering the number of opposing natural tooth pairs allowed further differentiation between Stage III and Stage IV. This is consistent with recent publications in which the number of cases upgraded from Stage II to Stage III periodontitis was low when complexity factors were considered (Eickholz et al. 2025; Tay et al. 2025). However, it should be noted that these studies only evaluated a subset of the complexity factors, which may have affected the classification of individuals. Nevertheless, since recording complexity factors in epidemiological studies is time‐consuming and costly, using a simplified version of the ACES framework could encourage its wider adoption in epidemiological research. Future research will show whether a simplified version of the staging process within the ACES framework, which only considers interdental CAL, is useful.

Thirdly, 9% of individuals in SHIP‐TREND‐0 were not classified. Compared to that in other studies (Dos Anjos et al. 2024; Eickholz et al. 2025; Meng et al. 2025; Tay et al. 2025), this proportion was found to be the highest. In SHIP‐TREND‐0, subjects were classified as ‘non‐classified’ if < 2 non‐adjacent teeth with available interdental CAL measurements were present, or if < 2 teeth had buccal or oral CAL and PD measurements (Holtfreter et al. 2024). In these cases, the CDC/AAP and EFP/AAP periodontitis case definitions could not be applied. It is important to note that CAL was not measured at crowned teeth in SHIP‐TREND‐0 and DMS * 6. Consequently, subjects with exclusively crowned teeth were categorised as non‐classified. The methodology employed in NHANES was different (Centers for Disease Control and Prevention 2009). When the margin of a restoration was below the CEJ or when calculus obscured the CEJ, it was estimated using adjacent landmarks and dental anatomy, as was the CAL. Consequently in NHANES, subjects with only crowned teeth were not categorised as non‐classified. These issues highlight the impact of methodology and classification criteria on prevalence estimates, emphasising the need for standardised protocols to ensure comparability across studies.

Fourthly, we investigated whether the risk of tooth loss increases significantly when the dentition collapses, as is generally observed in Stage IV cases (Tonetti et al. 2018). In SHIP‐TREND, 2.6% of Stage IV cases became edentulous during the seven‐year follow‐up period, as opposed to 0%–0.3% of Stage I to III cases. In line, the adjusted IRR for the number of extracted teeth increased markedly between Stage III (3.60) and Stage IV (8.69). Clinical studies have associated Stage IV periodontitis with high rates of edentulism (Graetz et al. 2019; Koffi‐Coulibaly et al. 2021). Notably, 15.2% of non‐classified cases also became edentulous. These cases had an average of 8.7 teeth at baseline and were characterized by bite collapse and a high risk of incident edentulism. This suggests that cases not classified according to the ACES framework may be consistent with Stage IV disease. These results highlight the need for complex and effective interventions in order to prevent tooth loss and edentulism.

Fifthly, we assessed whether the prevalence estimates agreed with the CDC/AAP classification. We observed a low level of agreement between the ACES framework and the CDC/AAP classification (see Figure 1). Although the prevalences of Stage IV (ACES; 17.4%) and severe periodontitis (CDC/AAP; 16.8%) seemed similar at first, Sankey graphs revealed considerable disparities between the two classifications. Notably, 38.6% of individuals classified as having no periodontitis by the CDC/AAP criteria were classified as having Stage I periodontitis and 44.1% were classified as having Stage II periodontitis. Moderate periodontitis corresponded to a certain extent with Stage II (23.6%) but more so with stages III (38.5%) and IV (32.9%). These transition patterns align well with recent publications (Dos Anjos et al. 2024; Eickholz et al. 2025; Tay et al. 2025). Conversely, when diagnostic performance of the ACES framework was assessed, which was methodologically accompanied by combining categories, which in turn results in information loss, the ACES framework (Stage III–IV) was highly sensitive in identifying moderate to severe cases. However, this could simply be explained by the CAL threshold being aligned (ACES: 3–4 mm; CDC/AAP: ≥ 3 mm). Notably, sensitivity of opposing Stage IV with ‘Healthy to Stage III’ for identifying severe cases dropped to 41.8%. This aligned well with previous studies (Dos Anjos et al. 2024; Tay et al. 2025). In summary, the differences between the two classifications are primarily influenced by the basic threshold for diagnosing periodontal disease and by the fact that interdental CAL dominates the staging process under the ACES framework. Furthermore, prevalence estimates from studies reporting only the CDC/AAP criteria cannot be compared with those reporting only the EFP/AAP classification.

This study has several strengths. SHIP‐TREND is a large‐scale, population‐based cohort spanning a wide age range and with a long follow‐up. The dental examiners received extensive training and calibration, and standardised protocols for data collection were employed to ensure high‐quality data recording. Among the study's limitations, the primary enrolment of healthier individuals with better compliance and greater health awareness at both baseline and follow‐up must be mentioned, shifting the prevalence towards lower stages. Second, enrolment of only Caucasian subjects limited the generalisability of the findings to other ethnic groups. Third, the prevalence of periodontitis was underestimated due to use of the half‐mouth protocol with four sites. Finally, not all of the complexity factors suggested by the ACES framework for completed studies were used. For instance, SHIP‐TREND did not collect data on furcation involvement and vertical bone loss.

## 5 Conclusions

Using the EFP/AAP classification and the ACES framework, the prevalence of periodontitis in SHIP‐TREND‐0 was found to be considerably high (above 78.6%), likely overestimating actual treatment needs. Given the methodological issues outlined above, the application of the EFP/AAP classification using the ACES framework and current interdental CAL thresholds to epidemiological data remains questionable. Furthermore, our results emphasised that prevalence estimates from the CDC/AAP classification and the ACES framework are not directly comparable. Despite these challenges, SHIP‐TREND provides valuable long‐term data that contribute to a deeper understanding of periodontitis prevalence and severity and its relation to incident tooth loss. Future research should further investigate the applicability of the ACES framework in diverse populations, using standardised protocols to improve comparability across studies.

## Author Contributions

S.N., T.K. and B.H. substantially contributed to the conception or design of the work. S.N., C.P., H.V., T.K., B.H. and P.K. contributed to the acquisition, analysis or interpretation of data. S.N., T.K. and B.H. drafted the manuscript. S.N., T.K., C.P., H.V., B.H. and P.K. revised the manuscript critically for important intellectual content. All authors approved the final version of the manuscript and are accountable for all aspects of the work.

## Funding

SHIP is part of the Community Medicine Research Network of the University Medicine Greifswald, which is supported by the German Federal State of Mecklenburg‐West Pomerania. C.P. was funded by the Deutsche Forschungsgemeinschaft (DFG, German Research Foundation) ‐ 451892213.

## Ethics Statement

SHIP‐TREND was positively evaluated by the ethics committee of the University of Greifswald (SHIP‐TREND‐0: BB 39/08a; SHIP‐TREND‐1: BB 174/15). All participants were informed about the study protocol and signed the informed consent and the privacy statement.

## Conflicts of Interest

The authors declare no conflicts of interest.

## Supporting information


**Data S1:** Supporting information.
**Table S1:** Overview on studies reporting the prevalence of periodontitis according to the European Federation of Periodontology/American Academy of Periodontology Classification (EFP/AAP) classification with or without the application of the ACES framework.
**Table S2:** Baseline characteristics of participants included in the cross‐sectional analyses (*N* = 3890) stratified by the gingivitis/periodontitis status according to the ACES framework (localised and generalised gingivitis cases were combined to retrieve reasonable sample sizes).
**Table S3:** Distribution of SHIP‐TREND‐0 participants (*N* = 3890) according to the ACES framework stratified by age group.
**Table S4:** Grade (according to Schumacher et al.) in periodontitis cases according to the ACES framework.
**Table S5:** Comparison of the CDC/AAP classification and the ACES framework (*N* = 3890).
**Table S6:** Cross tabulation of the ACES framework and the CDC/AAP classification (gold standard), excluding non‐classified and edentulous cases.
**Figure S1:** Flowchart for the selection of participants for cross‐sectional and longitudinal analyses. Of the 4420 baseline participants, 4321 had dental examinations and of those, 3890 had also periodontal examinations. After exclusion of participants without follow‐up examinations (*N* = 1510), edentulous subjects (*N* = 77) and subjects without tooth loss data at follow‐up (*N* = 9), 2294 participants remained for longitudinal analyses.

## Data Availability

The data that support the findings of this study are available from Forschungsverbund Community Medicine. Restrictions apply to the availability of these data, which were used under license for this study. Data are available from https://transfer.ship‐med.uni‐greifswald.de/FAIRequest/login with the permission of Forschungsverbund Community Medicine.
