# Supplementary material for: Prevalence of Periodontitis and Methodological Aspects of the 2018 EFP/AAP Classification—The Study of Health in Pomerania (SHIP‐TREND)
Source: J Clin Periodontol. 2026 Apr 29;53(7):1046–55. doi: 10.1111/jcpe.70137 (PMC13263721; doi:10.1111/jcpe.70137)
Supplement: Supplementary file 1 — Data S1: Supporting information. Table S1: Overview on studies reporting the prevalence of periodontitis according to the European Federation of Periodontology/American Academy of Periodontology Classification (EFP/AAP) classification with or without the application of the ACES framework. Table S2: Baseline characteristics of participants included in the cross‐sectional analyses (N = 3890) stratified by the gingivitis/periodontitis status according to the ACES framework (localised and generalised gingivitis cases were combined to retrieve reasonable sample sizes). Table S3: Distribution of SHIP‐TREND‐0 participants (N = 3890) according to the ACES framework stratified by age group. Table S4: Grade (according to Schumacher et al.) in periodontitis cases according to the ACES framework. Table S5: Comparison of the CDC/AAP classification and the ACES framework (N = 3890). Table S6: Cross tabulation of the ACES framework and the CDC/AAP classification (gold standard), excluding non‐classified and edentulous cases. Figure S1: Flowchart for the selection of participants for cross‐sectional and longitudinal analyses. Of the 4420 baseline participants, 4321 had dental examinations and of those, 3890 had also periodontal examinations. After exclusion of participants without follow‐up examinations (N = 1510), edentulous subjects (N = 77) and subjects without tooth loss data at follow‐up (N = 9), 2294 participants remained for longitudinal analyses. [file JCPE-53-1046-s001.docx]

Supplemental Material

**Prevalence of periodontitis and methodological aspects of the 2018 EFP/AAP classification – the Study of Health in Pomerania (SHIP-TREND)**

Sonya Nafz, Thomas Kocher, Christiane Pink, Henry Völzke, Philipp Kanzow, Birte Holtfreter

**Material and methods**

*Study participants*

Of the 4,420 baseline participants, 4,321 had dental examinations and of those, 3,890 had also periodontal examinations (Appendix Figure 1). After exclusion of participants without follow-up examinations (N=1,510), edentulous subjects (N=77), and subjects without tooth loss data at follow-up (N=9), 2,294 participants remained for longitudinal analyses. The 7-year follow-up (SHIP-TREND-1) was conducted from 2016 to 2019, including 2,507 participants, with an average follow-up of 7.4 years.

*Periodontal examination*

PD and CAL were measured at distobuccal, midbuccal, mesiobuccal, and midlingual/midpalatinal sites according to the half-mouth method excluding third molars (left or right side randomly selected) using a manual periodontal probe (PCPUNC 15, Hu-Friedy, Chicago, IL, USA). Measurements were mathematically rounded to the next whole millimetre. PD was measured as the distance between free gingival margin (FGM) and pocket base. If the cemento–enamel junction (CEJ) was located sub-gingivally, CAL was calculated as PD minus the distance between FGM and CEJ. If recession was present at the examined site, CAL was directly measured as the distance between CEJ and the pocket base. Where the determination of the CEJ was indistinct (wedge-shaped defects, fillings, and crown margins), CAL was not recorded. Bleeding on probing (BOP) was recorded at the identical four sites on the first incisor, the canine and the first molar in each probed quadrant. If teeth were missing, the next distally located tooth was assessed.

*Periodontal variables and classifications*

On participant level, the percentage of bleeding sites (BOP), mean PD, the percentage of sites with PD ≥4 mm, and mean CAL and mean interdental CAL were calculated. Participants were also classified according to the Centers for Disease Control and Prevention/American Academy of Periodontology (CDC/AAP) case definition of periodontitis (Eke et al., 2012). Participants were classified as having no, mild, moderate, or severe periodontitis or being a non-classified case. Finally, the number of missing teeth was calculated (excluding third molars).

We implemented the EFP/AAP classification schemes for gingivitis (Chapple et al., 2018) and periodontitis (Papapanou et al., 2018) using the ACES framework for completed studies (Holtfreter et al., 2024). Accordingly, the following variables were derived: maximum PD; number of remaining natural teeth in the upper and lower quadrant; number of opposing pairs of natural teeth (counting also positions with gap closure as ‘present’); having ≥2 non-adjacent teeth with interdental CAL measurements; number of non-adjacent teeth with maximum PD ≥6 mm; number of teeth with buccal or oral CAL and PD measurements; having interdental CAL ≥1 mm at ≥2 non-adjacent teeth; presence of buccal or oral CAL ≥3mm with PD >3mm at ≥2 teeth; maximum interdental CAL per tooth; percentage of teeth with CAL 1-2, 3-4, or ≥5 mm.

Participants were classified as follows: edentulous, periodontal health, localized gingivitis, generalized gingivitis, periodontitis cases, and non-classified cases. The non-classified cases are listed here according to the above-mentioned criteria of the ACES framework. This case definition is not included in either classification. Periodontitis cases were staged based on maximum interdental CAL, PDs (PD ≥6 mm at ≥2 non-adjacent teeth, disregarding PDs at third molars and at distal surfaces of second molars) and the number of opposing pairs of natural teeth (<10 versus ≥10, excluding third molars). The following complexity factors were not considered: furcation involvement, vertical bone loss, tooth mobility, and bite collapse/drifting/flaring.

Grade was determined as suggest by (Papapanou et al., 2018), using indirect evidence of progression, which can be inferred by considering participant’s age and relative CAL as a percentage of the root length (Holtfreter et al., 2024). Root length data were derived from two studies (Salonen et al., 1991; Schumacher et al., 1995). While Salonen et al. (1991) provided sex-specific mesial and distal measurements for 732 randomly selected Swedish adults for all teeth, Schumacher (1995) only provided standard root length for all teeth (excluding third molars) without distinction between sexes. Extent was defined as the percentage of teeth affected at the stage-defining severity level (Holtfreter et al., 2024).

Under the following link, information and syntax for the implementation of the ACES framework in SHIP-TREND-0 is available: https://github.com/ACES-periodontitis.

*Calibration data*

In SHIP-TREND-0, dental examinations were conducted by six calibrated examiners. In calibration exercises, all dentists repeatedly examined five persons not connected to the study. Intra-rater correlations for CAL measurements ranged between 0.67 and 0.89 and inter-rater correlation was 0.70. For PD measurements, the examiners yielded intra-rater correlations between 0.68 and 0.88 and an inter-rater correlation of 0.72. For assessment of the tooth status, Cohen’s kappa reliability coefficients were 0.93-0.99 (intra-examiner) and 0.94-0.98 (pairwise inter-examiner).

In SHIP-TREND-1, dental examinations were conducted by six calibrated examiners. In calibration exercises, all dentists repeatedly examined five persons not connected to the study. For assessment of the tooth status, Cohen’s kappa reliability coefficients were 0.97-1.00 (intra-examiner) and 0.91-0.96 (pairwise inter-examiner).

*Covariates*

From the computer-assisted personal interview the following items were retrieved. School education was defined as <10, 10, >10 years. Smoking was categorized as never, former, and current smoking. Self-reported dental visit within the last 12 months and daily use of interdental cleaning aids were recorded. Powered tooth brush usage was defined opposing ‘neither manual (MTB) nor powered tooth brush usage (PTB)’ with ‘MTB only’, ‘PTB and MTB’, and ‘PTB only’. Based on the toothbrushing frequency, participants were categorized as ‘<2 times/day’ or ‘≥2 times/day’. Standardized measurements of body height and weight were performed with calibrated scales and the body mass index (BMI) was calculated as weight divided by height squared (kg/m²). Known diabetes mellitus was defined as physician's diagnosis or antidiabetic medication intake (Anatomic Therapeutic Chemical classification system; code A10). Fasting blood samples were drawn from the cubital vein in the supine position and aliquots were prepared for immediate analysis and for storage at −80 °C. Haemoglobin A1c (HbA1c) concentrations were determined by high-performance liquid chromatography (Bio-Rad Diamat, Munich, Germany). The follow-up time was indicated in exact years.

**References**

Chapple, I. L. C., Mealey, B. L., Van Dyke, T. E., Bartold, P. M., Dommisch, H., Eickholz, P., . . . Yoshie, H. (2018). Periodontal health and gingival diseases and conditions on an intact and a reduced periodontium: Consensus report of workgroup 1 of the 2017 World Workshop on the Classification of Periodontal and Peri-Implant Diseases and Conditions. *J Clin Periodontol, 45 Suppl 20*, S68-S77. doi:10.1111/jcpe.12940

Eke, P. I., Page, R. C., Wei, L., Thornton-Evans, G., & Genco, R. J. (2012). Update of the case definitions for population-based surveillance of periodontitis. *J Periodontol, 83*(12), 1449-1454. doi:10.1902/jop.2012.110664

Holtfreter, B., Kuhr, K., Borof, K., Tonetti, M. S., Sanz, M., Kornman, K., . . . Papapanou, P. N. (2024). ACES: A new framework for the application of the 2018 periodontal status classification scheme to epidemiological survey data. *J Clin Periodontol, 51*(5), 512-521. doi:10.1111/jcpe.13965

Papapanou, P. N., Sanz, M., Buduneli, N., Dietrich, T., Feres, M., Fine, D. H., . . . Tonetti, M. S. (2018). Periodontitis: Consensus report of workgroup 2 of the 2017 World Workshop on the Classification of Periodontal and Peri-Implant Diseases and Conditions. *J Clin Periodontol, 45 Suppl 20*, S162-S170. doi:10.1111/jcpe.12946

Salonen, L. W., Frithiof, L., Wouters, F. R., & Hellden, L. B. (1991). Marginal alveolar bone height in an adult Swedish population. A radiographic cross-sectional epidemiologic study. *J Clin Periodontol, 18*(4), 223-232. doi:10.1111/j.1600-051x.1991.tb00419.x

Schumacher, G.-H., & Gente, M. (1995). *Odontographie - Anatomie der Zähne und des Gebisses* (Vol. 5th edition). Heidelberg, Germany: Huethig GmbH.

Table S1. Overview on studies reporting the prevalence of periodontitis according to the European Federation of Periodontology/American Academy of Periodontology Classification (EFP/AAP) classification with or without the application of the ACES framework.

| Author, year | Country or region | Data collection period | Study name | Study design | Number of participants | Age | Sex | Protocol | Complexity factors | ACES framework | Stage I  prevalence | Stage II  prevalence | Stage III  prevalence | Stage IV  prevalence |
| --- | --- | --- | --- | --- | --- | --- | --- | --- | --- | --- | --- | --- | --- | --- |
| Jiao et al., 2021 | Mainland China | 2015 to 2016 | Fourth National Oral Health Survey | cross-sectional | n=13,459 | 35 – 74 | M: 49.8%  F: 50.2% | (6/tooth) full-mouth | PD, number of remaining teeth and opposing pairs | ‍no | 15.5% | 16.3% | 15.7% | 14.9% |
| Meng et al., 2024 | America | 2011 to 2014 | National Health and Nutrition Examination Survey (NHANES) | cross-sectional | n=7,651 | ≥30 | M: 49.0%  F: 51.0% | (6/tooth) full-mouth | PD, number of opposing pairs of natural teeth | yes | 13.9% | 50.6% | 15.6% | 12.8% |
| Tay et al., 2025 | America | 2009 to 2014 | National Health and Nutrition Examination Survey (NHANES) | cross-sectional | n=11,686 | ≥30 | NA | (6/tooth) full-mouth | PD, number of opposing pairs of natural teeth | ‍yes | 17.9% | 46.2% | 16.7% | 12.4% |
| Stødle et al., 2021 | County of Nord-Trøndelag, Norway | 2017 to 2019 | The Trøndelag Health Study (Helseundersøkelsen i Trøndelag 4, HUNT4) | cross-sectional | n=4,863 | 19 – 94 | M: 44.1%  F: 55.9% | (6/tooth) full-mouth | PD, vertical bone loss, furcation grade, bite collapse/drifting/flaring | ‍no | 13.8% | 41.1% | 15.3% | 2.3% |
| Holde, Bunæs, & Jönsson, 2025 | Tromsø, Norway | 2015 to 2016 | Tromsø7 | cross-sectional | n=3,701 | 40 – 99 | M: 48.2%  F: 51.8% | (6/tooth) full-mouth | PD | ‍no | 23.5% | 44.9% | 20.9% * | NA |
| Ortigara et al., 2021 | Rural area of Rosário do Sul, southern Brazil | 2015 to 2016 | NA | cross-sectional | n=588 | 15 – 93 | M: 50.3%  F: 49.7% | (6/tooth) full-mouth | PD and number of remaining teeth were collected but not explicitly mentioned for use in the classification | ‍no | 2.7% | 26.2% | 71.1%* | NA |
| Dos Anjos et al., 2024 | Curitiba, Paraná, Brazil | 2020 to 2022 | NA | cross-sectional | N=555 | ≥18 | M: 40.8%  F: 59.2% | (6/tooth) full-mouth | PD, number remaining natural teeth | yes | 5.3% | 29.0% | 24.0% | 19.3% |
| Morales et al., 2022 | Chile | 2007 to 2008 | First Chilean National Examination Survey | cross-sectional | n=1,087  n=369 | 35 – 44  65 – 74 | M: 44.1%  F: 55.9% | (6/tooth) full-mouth | PD, number of opposing pairs of natural teeth | ‍no | 0.1% | 4.7% | 12.8% | 81.3% |
| Eickholz et al., 2025 | Germany | 2021 to 2023 | The 6th German Oral Health Study (DMS • 6) | cross-sectional | n=912  n=755 | 35 – 44  65 – 74 | M: 49.7%  F: 50.2%  M: 46.1%  F: 53.9% | (6/tooth) full-mouth | PD, flaring of maxillary anterior teeth, and the number of remaining and occluding pairs of natural teeth | ‍yes | 31.6%  8.3% | 46.0%  24.2% | 13.6%  26.3% | 3.9%  26.4% |
| Garcia, de la Vega, & Yanga-Mabunga, 2024 | Four identified geographical regions in the Philippines | 2018 to 2019 | substudy of FITforFRAIL | cross-sectional | n=183 | ≥60 | M: 39.9%  F: 60.1% | (6/tooth) full-mouth | PD and number of remaining teeth were collected but not explicitly mentioned for use in the classification | ‍no | 0 | 5.5% | 94.5%* | NA |

*Note*: * reported as Stage III/IV prevalence. Abbreviations: F, females; M, males; PD, probing depth; n, number of subjects; NA, not available.

Table S2. Baseline characteristics of participants included in the cross-sectional analyses (N=3,890) stratified by the gingivitis/periodontitis status according to the ACES framework (localized and generalized gingivitis cases were combined to retrieve reasonable sample sizes).

|  | N | Total | Edentulous  N=269 | Periodontal health  N=88 | Gingivitis  N=90 | Stage I  N=522 | Stage II  N=1104 | Stage III  N=729 | Stage IV  N=716 | Non-classified  N=372 |
| --- | --- | --- | --- | --- | --- | --- | --- | --- | --- | --- |
| Age, years | 3,890 | 51.3±15.2  52 (39; 63) | 68.1±8.8  70 (61; 75) | 31.6±8.4  29 (25; 36) | 34.1±11.6  31 (25; 40) | 37.2±11.1  35 (29; 43) | 45.7±12.6  45 (36; 55) | 52.1±11.8  51 (44; 61) | 60.9±10.4  61 (53; 69) | 64.4±9.8  66 (58; 72) |
| Male sex | 3,890 | 1,915 (49.2%) | 150 (55.8%) | 33 (37.5%) | 44 (48.9%) | 210 (40.2%) | 491 (44.5%) | 418 (57.3%) | 398 (55.6%) | 171 (46.0%) |
| School education | 3,882 |  |  |  |  |  |  |  |  |  |
| <10 years |  | 833 (21.5%) | 166 (61.7%) | 2 (2.3%) | 10 (11.1%) | 32 (6.1%) | 142 (12.9%) | 105 (14.4%) | 240 (33.7%) | 136 (37.0%) |
| 10 years |  | 2,021 (52.1%) | 78 (29.0%) | 44 (50.0%) | 53 (58.9%) | 290 (55.6%) | 629 (57.0%) | 418 (57.3%) | 347 (48.7%) | 162 (44.0%) |
| >10 years |  | 1,028 (26.5%) | 25 (9.3%) | 42 (47.7%) | 27 (30.0%) | 200 (38.3%) | 332 (30.1%) | 206 (28.3%) | 126 (17.7%) | 70 (19.0%) |
| Living in a partnership, yes | 3,882 | 3,031 (78.1%) | 189 (70.3%) | 58 (65.9%) | 58 (64.4%) | 393 (75.3%) | 870 (78.9%) | 607 (83.3%) | 574 (80.5%) | 282 (76.6%) |
| Smoking status | 3,881 |  |  |  |  |  |  |  |  |  |
| Never |  | 1,410 (36.3%) | 71 (26.4%) | 35 (39.8%) | 31 (34.4%) | 216 (41.4%) | 424 (38.4%) | 256 (35.1%) | 246 (34.5%) | 131 (35.7%) |
| Former |  | 1,430 (36.9%) | 119 (44.2%) | 29 (33.0%) | 22 (24.4%) | 162 (31.0%) | 371 (33.6%) | 269 (36.9%) | 303 (42.5%) | 155 (42.2%) |
| Current, <10 cigarettes/day |  | 218 (5.6%) | 10 (3.7%) | 9 (10.2%) | 11 (12.2%) | 43 (8.2%) | 65 (5.9%) | 35 (4.8%) | 35 (4.9%) | 10 (2.7%) |
| Current, ≥10 cigarettes/day |  | 701 (18.1%) | 66 (24.5%) | 10 (11.4%) | 22 (24.4%) | 70 (13.4%) | 195 (17.7%) | 151 (20.7%) | 120 (16.8%) | 67 (18.3%) |
| Current, missing |  | 122 (3.1%) | 3 (1.1%) | 5 (5.7%) | 4 (4.4%) | 31 (5.9%) | 48 (4.4%) | 18 (2.5%) | 9 (1.3%) | 4 (1.1%) |
| Body Mass Index, kg/m^2^ | 3,885 | 28.0±5.1  27.4 (24.4; 30.9) | 30.4±5.5  30.0 (26.7; 33.4) | 24.5±4.2  24.1 (21.5; 26.7) | 27.0±5.4  26.3 (22.7; 31.3) | 25.8±4.7  25.0 (22.3; 28.2) | 27.2±4.8  26.6 (23.9; 30.0) | 28.0±4.6  27.5 (24.6; 30.8) | 29.6±5.4  28.9 (26.0; 32.4) | 29.5±5.1  28.9 (26.4; 31.9) |
| Haemoglobin A1c, % | 3,883 | 5.3±0.8  5.2 (4.9; 5.6) | 6.0±1.2  5.7 (5.3; 6.3) | 4.8±0.5  4.8 (4.6; 5.1) | 4.9±0.5  4.8 (4.7; 5.1) | 5.0±0.7  5.0 (4.7; 5.3) | 5.2±0.7  5.1 (4.8; 5.5) | 5.4±0.7  5.3 (5.0; 5.6) | 5.6±0.9  5.4 (5.1; 5.9) | 5.6±0.9  5.5 (5.1; 5.9) |
| Known type 2 diabetes mellitus, yes | 3,885 | 378 (9.7%) | 75 (27.9%) | 2 (2.3%) | 1 (1.1%) | 13 (2.5%) | 51 (4.6%) | 56 (7.7%) | 105 (14.7%) | 75 (20.3%) |
| Haemoglobin A1c ≥7% in individuals with known type 2 diabetes, yes | 378 | 121 (32.0%) | 7.0±1.3  6.9 (6.2; 7.6) | 6.0±1.8  6.0 (4.7; 7.2) | 4.5 (-)  4.5 (4.5; 4.5) | 6.2±2.1  5.3 (5.0; 6.9) | 6.1±1.2  6.0 (5.3; 6.8) | 6.4±1.1  6.2 (5.8; 7.2) | 6.7±1.4  6.5 (5.9; 7.3) | 6.5±1.1  6.3 (5.8; 7.1) |
| Daily use of interdental cleaning aids, yes | 3,830 | 1,023 (26.7%) | - | 19 (21.6%) | 19 (21.1%) | 111 (21.3%) | 301 (27.3%) | 259 (35.5%) | 243 (33.9%) | 70 (18.8%) |
| Tooth brushing frequency | 3,830 |  |  |  |  |  |  |  |  |  |
| <2 times daily |  | 593 (15.5%) | 53 (25.4%) | 6 (6.8%) | 20 (22.2%) | 72 (13.8%) | 147 (13.3%) | 109 (15.0%) | 118 (16.5%) | 68 (18.3%) |
| ≥2 times daily |  | 3,237 (84.5%) | 156 (74.6%) | 82 (93.2%) | 70 (77.8%) | 450 (86.2%) | 57 (86.7%) | 620 (85.0%) | 598 (83.5%) | 304 (81.7%) |
| Tooth brush type | 3,830 |  |  |  |  |  |  |  |  |  |
| Neither MTB nor PTB |  | 43 (1.1%) | - | 0 (0%) | 0 (0%) | 0 (0%) | 2 (0.2%) | 4 (0.5%) | 7 (1.0%) | 5 (1.3%) |
| MTB |  | 2,816 (73.5%) | - | 59 (67.1%) | 65 (72.2%) | 338 (64.7%) | 752 (68.1%) | 505 (69.3%) | 589 (82.3%) | 327 (87.9%) |
| PTB |  | 740 (19.3%) | - | 20 (22.7%) | 15 (16.7%) | 145 (27.8%) | 269 (24.4%) | 174 (23.9%) | 90 (12.6%) | 24 (6.5%) |
| MTB and PTB |  | 231 (6.0%) | - | 9 (10.2%) | 10 (11.1%) | 39 (7.5%) | 81 (7.3%) | 46 (6.3%) | 30 (4.2%) | 16 (4.3%) |
| Dental visit within the last 12 months, yes | 3,830 | 3,402 (88.8%) | 149 (71.3%) | 78 (88.6%) | 75 (83.3%) | 465 (89.1%) | 995 (90.1%) | 668 (91.6%) | 650 (90.8%) | 322 (86.6%) |
| Self-reported periodontal treatment within the last 5 years, yes |  | 703 (18.5%) | 11 (5.3%) | 3 (3.5%) | 8 (8.9%) | 48 (9.2%) | 168 (15.4%) | 220 (30.3%) | 172 (24.1%) | 73 (19.9%) |
| Percentage of sites with bleeding on probing, % | 3,618 | 25.3±24.4  18.8 (6.3; 37.5) | - | 3.9±3.3  4.2 (0; 8.3) | 22.1±10.2  20.8 (15.0; 29.2) | 13.8±16.3  8.3 (0; 20.8) | 19.3±19.0  15.0 (4.2; 29.2) | 29.7±23.4  25.0 (12.5; 41.7) | 35.7±27.4  30.0 (12.5; 50.0) | 37.0±31.7  25.0 (10.0; 60.0) |
| Mean PD, mm | 3,621 | 2.59±0.71  2.40 (2.14; 2.83) | - | 2.04±0.21  2.03 (1.90; 2.16) | 2.21±0.30  2.19 (2.02; 2.35) | 2.12±0.24  2.13 (1.96; 2.27) | 2.30±0.31  2.27 (2.09; 2.48) | 2.81±0.60  2.69 (2.39; 3.10) | 3.02±0.82  2.85 (2.46; 3.41) | 3.07±1.05  2.85 (2.38; 3.50) |
| Percentage of sites with PD ≥4 mm, % | 3,621 | 14.5±19.4  6.3 (0; 20.5) | - | 1.5±2.6  0 (0; 2.0) | 4.7±7.7  2.1 (0; 5.8) | 1.7±4.2  0 (0; 1.8) | 5.6±7.6  2.8 (0; 7.7) | 21.4±18.2  16.7 (7.7; 30.4) | 27.3±22.9  21.4 (8.3; 40.6) | 25.6±27.3  16.2 (0; 43.3) |
| Mean CAL, mm | 3,432 | 2.47±1.70  2.07 (1.27; 3.33) | - | 0.24±0.28  0.18 (0.05; 0.35) | 0.32±0.48  0.17 (0.05; 0.38) | 1.03±0.37  1.07 (0.81; 1.27) | 1.71±0.61  1.69 (1.29; 2.09) | 2.90±1.05  2.81 (2.18; 3.50) | 4.23±1.54  4.00 (3.13; 5.08) | 4.76±2.32  4.38 (2.88; 6.13) |
| Mean interdental CAL, mm | 3,430 | 2.56±1.80  2.17 (1.29; 3.50) | - | 0.05±0.15  0 (0; 0.07) | 0.16±0.45  0 (0; 0.17) | 1.04±0.43  1.04 (0.93; 1.35) | 1.76±0.67  1.76 (1.31; 2.21) | 3.07±1.16  3.00 (2.31; 3.78) | 4.38±1.63  4.17 (3.20; 5.25) | 4.91±2.42  4.50 (3.00; 6.25) |
| CDC/AAP classification | 3,347 |  |  |  |  |  |  |  |  |  |
| No periodontitis |  | 1,337 (40.0%) | - | 85 (96.6%) | 83 (92.2%) | 516 (98.9%) | 590 (53.4%) | 21 (2.9%) | 19 (2.6%) | 23 (23.5%) |
| Mild periodontitis |  | 279 (8.3%) | - | 0 (0%) | 3 (3.3%) | 0 (0%) | 238 (21.6%) | 17 (2.3%) | 14 (2.0%) | 7 (7.1%) |
| Moderate periodontitis |  | 1,167 (34.9%) | - | 3 (3.4%) | 4 (4.4%) | 6 (1.1%) | 276 (25.0%) | 449 (61.6%) | 384 (53.6%) | 45 (45.9%) |
| Severe periodontitis |  | 564 (16.8%) | - | 0 (0%) | 0 (0%) | 0 (0%) | 0 (0%) | 242 (33.2%) | 299 (41.8%) | 23 (23.5%) |
| Number of teeth | 3,890 | 20.2±8.6  24 (17; 27) | 0 (-) | 26.9±2.0  28 (26; 28) | 25.5±3.8  27 (25; 28) | 25.8±3.1  27 (25; 28) | 24.5±3.6  25 (23; 27) | 25.3±2.0  26 (24; 27) | 16.5±5.1  18 (13; 21) | 8.2±5.4  7 (4; 11) |

Data are presented as means ± standard deviations and medians (25%; 75% quantiles) or as numbers (percentages). Abbreviations: AAP, American Academy of Periodontology; CAL, clinical attachment level; CDC, Centers for Disease Control and Prevention; MTB, manual tooth brush; PD, probing depth; PTB, powered tooth brush.

Table S3. Distribution of SHIP-TREND-0 participants (N=3,889) according to the ACES framework stratified by age group.

|  | 20-24 years | 25-34 years | 35-44 years | 45-54 years | 55-64 years | 65-74 years | 75-84 years |
| --- | --- | --- | --- | --- | --- | --- | --- |
| Edentulous | 0% (-) | 0% (-) | 0.3% (0.20) | 1.8% (0.46) | 8.8% (1.02) | 17.8% (1.59) | 30.9% (3.20) |
| Periodontal health | 15.2% (3.24) | 9.3% (1.43) | 2.5% (0.60) | 0.5% (0.24) | 0.2% (0.17) | 0% (-) | 0% (-) |
| Localized gingivitis | 12.8% (3.16) | 5.6% (1.04) | 2.3% (0.53) | 0.5% (0.28) | 0.3% (0.23) | 0.3% (0.19) | 0% (-) |
| Generalized gingivitis | 1.9% (1.37) | 1.5% (0.54) | 0.3% (0.24) | 0.3% (0.18) | 0.4% (0.22) | 0% (-) | 0% (-) |
| Periodontitis cases | 70.1% (4.17) | 83.3% (1.75) | 93.7% (0.90) | 90.3% (1.04) | 76.9% (1.51) | 60.3% (1.99) | 43.3 (3.48) |
| Stage I | 45.7% (4.64) | 36.6% (2.26) | 22.0% (1.60) | 8.4% (0.99) | 3.8% (0.70) | 2.0% (0.54) | 0.4% (0.41) |
| Stage II | 19.2% (3.61) | 38.3% (2.27) | 44.0% (1.91) | 34.1% (1.69) | 22.7% (1.49) | 11.2% (1.23) | 5.5% (1.63) |
| Stage III | 5.2% (2.15) | 7.4% (1.11) | 22.2% (1.62) | 27.8% (1.61) | 22.1% (1.48) | 15.1% (1.44) | 6.9% (1.71) |
| Stage IV | 0% (-) | 1.0% (0.47) | 5.5% (0.91) | 20.0% (1.42) | 28.3% (1.63) | 32.1% (1.90) | 30.5% (3.24) |
| Non-classified cases | 0% (-) | 0.3% (0.21) | 0.9% (0.33) | 6.6% (0.88) | 13.4 (1.23) | 21.6% (1.69) | 25.8% (3.01) |
| Total number of subjects | 121 (100%) | 531 (100%) | 707 (100%) | 819 (100%) | 815 (100%) | 664 (100%) | 233 (100%) |

Data are given as percentages with standard errors in brackets. Survey-weighted estimates are reported. Abbreviations: ACES, Application of the 2018 Periodontal Status Classification to Epidemiological Survey Data.

Table S4. Grade (according to Schumacher et al.) in periodontitis cases according to the ACES framework.

| Stage | N | Grade A | Grade B | Grade C |
| --- | --- | --- | --- | --- |
| Stage I | 522 | 2.0% (0.56) | 80.8% (1.89) | 17.3% (1.84) |
| Stage II | 1,104 | 0% (-) | 74.4% (1.41) | 25.6% (1.41) |
| Stage III | 729 | 0% (-) | 47.3% (1.89) | 52.7% (1.89) |
| Stage IV | 716 | 0% (-) | 53.8% (1.90) | 46.2% (1.90) |

*Note:* Data are given as percentages with standard errors in brackets. Survey-weighted estimates are reported. Abbreviations: ACES, Application of the 2018 Periodontal Status Classification to Epidemiological Survey Data.

Table S5. Comparison of the CDC/AAP classification and the ACES framework (N=3,890).

| CDC/AAP classification | Periodontal  health | Localized gingivitis | Generalized  gingivitis | Stage I | Stage II | Stage III | Stage IV | Non-classified cases | Edentulous |
| --- | --- | --- | --- | --- | --- | --- | --- | --- | --- |
| No | 85 (6.4%) | 71 (5.3%) | 12 (0.9%) | 516 (38.6%) | 590 (44.1%) | 21 (1.6%) | 19 (1.4%) | 23 (1.7%) | 0 (0%) |
| Mild | 0 (0%) | 2 (0.7%) | 1 (0.4%) | 0 (0%) | 238 (85.3%) | 17 (6.1%) | 14 (5.0%) | 7 (2.5%) | 0 (0%) |
| Moderate | 3 (0.3%) | 0 (0%) | 4 (0.3%) | 6 (0.5%) | 276 (23.6%) | 449 (38.5%) | 384 (32.9%) | 45 (3.9%) | 0 (0%) |
| Severe | 0 (0%) | 0 (0%) | 0 (0%) | 0 (0%) | 0 (0%) | 242 (42.9%) | 299 (53.0%) | 23 (4.1%) | 0 (0%) |
| Edentulous | 0 (0%) | 0 (0%) | 0 (0%) | 0 (0%) | 0 (0%) | 0 (0%) | 0 (0%) | 0 (0%) | 269 (100%) |
| Non-classified | 0 (0%) | 0 (0%) | 0 (0%) | 0 (0%) | 0 (0%) | 0 (0%) | 0 (0%) | 274 (100%) | 0 (0%) |

*Note:* Data are presented as unweighted numbers (percentages). Abbreviations: AAP, American Academy of Periodontology; ACES, Application of the 2018 Periodontal Status Classification to Epidemiological Survey Data; CDC, Centers for Disease Control and Prevention.

Table S6. Cross tabulation of the ACES framework and the CDC/AAP classification (gold standard), excluding non-classified and edentulous cases.

| ***Identification of moderate/severe cases (CDC/AAP) by the ACES framework*** | | | |
| --- | --- | --- | --- |
|  | CDC/AAP classification | |  |
| ACES framework | No/mild | Moderate/severe | *Total* |
| Healthy to Stage II | 1,515 | 289 | *1,804* |
| Stage III to IV | 71 | 1,374 | *1,445* |
| *Total* | *1,586* | *1,663* | *3,249* |
| ***Identification of severe cases (CDC/AAP) by the ACES framework*** | | | |
| ACES framework | No/mild/moderate | Severe | *Total* |
| Healthy to Stage III | 1,515 | 289 | *1,804* |
| Stage IV | 71 | 1,374 | *1,445* |
| *Total* | *1,586* | *1,663* | *3,249* |

Note: Data are presented as unweighted numbers (percentages). Abbreviations: AAP, American Academy of Periodontology; ACES, Application of the 2018 Periodontal Status Classification to Epidemiological Survey Data; CDC, Centers for Disease Control and Prevention.


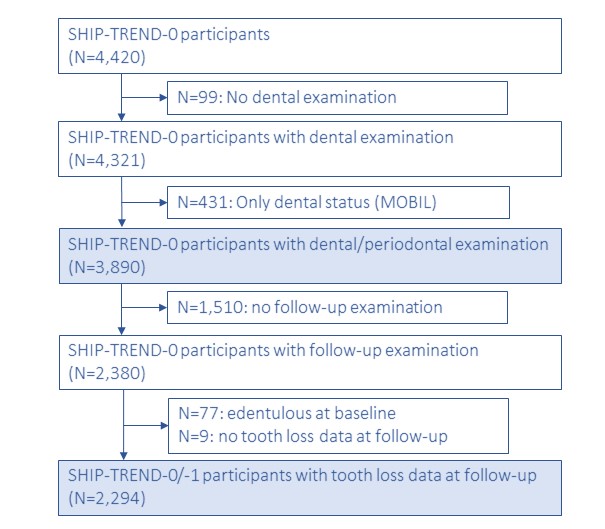


Figure S1. Flowchart for the selection of participants for cross-sectional and longitudinal analyses. Of the 4,420 baseline participants, 4,321 had dental examinations and of those, 3,890 had also periodontal examinations. After exclusion of participants without follow-up examinations (N=1,510), edentulous subjects (N=77), and subjects without tooth loss data at follow-up (N=9), 2,294 participants remained for longitudinal analyses.
